# Giant inguinal hernia with mal-rotation in a resource-limited area: Case report

**DOI:** 10.1016/j.ijscr.2025.110947

**Published:** 2025-01-27

**Authors:** Abdirahman Burale, Mahir Yusuf Kahir, Musse Ahmed, Ahmednour Sh Abdirahman Elmi, Abdirahman Ibrahim Said, Hassan Sh. Abdirahman Elmi

**Affiliations:** a Institute of Health Science, Jigjiga University, Ethiopia; b Borama Regional Hospital, Somalia; c College of Health Sciences, Amoud University, Somalia; d School of postgraduate, Amoud University, Somalia; e Faculty of Science, Charles University, Czechia

## Abstract

**Introduction:**

Giant inguinoscrotal hernias (GIH), defined as hernias extending below the inner thigh midpoint in a standing position, are rare and often seen in resource-limited settings due to delayed medical care. These hernias pose surgical challenges, particularly in low- and middle-income countries (LMICs), where standardized management protocols are lacking, and risks such as cardiorespiratory compromise are significant.

**Case presentation:**

A 55-year-old male presented with a large, irreducible right inguinoscrotal hernia of 1.5 years duration. Elective surgery involved sac separation and laparotomy, revealing herniation of bowel segments, including the terminal ileum and sigmoid colon, with concurrent intestinal malrotation. Ladd's procedure, appendectomy, and hernia repair were performed without complications. Postoperative recovery was uneventful, with the patient remaining asymptomatic during follow-up.

**Discussion:**

GIH management depends on hernia classification. While Type I hernias require simpler repairs, Types II and III often necessitate advanced techniques, such as Preoperative Progressive Pneumoperitoneum or bowel resection, to prevent abdominal compartment syndrome (ACS). Anatomical anomalies, such as malrotation, complicate surgical planning. Successful outcomes rely on individualized, resource-appropriate strategies and meticulous care, especially in LMICs.

**Conclusion:**

GIH presents unique challenges, particularly in resource-constrained settings. Tailored approaches, informed by classification and patient-specific factors, are essential. This case underscores the importance of innovative strategies, careful planning, and standardized protocols to improve outcomes for GIH patients globally.

## Highlights

• Call for Standardized GIH Management Protocol in low- and middle-income countries.
• Surgical Challenges in a Resource-Limited Setting.
• Tailored Surgical Strategy with Positive Outcome.

## 1 Introduction

Giant inguinoscrotal hernias are defined as hernias extending below the midpoint of the inner thigh in the standing position [1]. These hernias, though rare, often result from prolonged neglect or fear of surgical intervention and are more prevalent in rural populations and resource-limited regions, particularly in Sub-Saharan Africa [2]. In these low-resource settings, patients may avoid seeking medical care due to limited access, financial constraints, or lack of awareness, leading to the progression of hernias to this advanced stage.

These massive hernias present significant surgical challenges. One primary concern during the surgical repair is the potential for cardiorespiratory compromise, which can result from the sudden increase in intra-abdominal pressure following the replacement of herniated viscera into the abdominal cavity [3]. This issue underscores the need for careful preoperative assessment and planning in cases of giant hernias, especially in low- and middle-income countries (LMICs).

Unlike high-income countries (HICs), which are less likely to encounter such extensive hernias, LMICs face unique challenges in addressing these conditions. Given the frequency and complexity of these cases, preparedness for managing giant inguinoscrotal hernias is essential in LMICs [4]. Furthermore, there is currently no established standard treatment protocol for managing giant inguinoscrotal hernias, adding to the complexity and variability in their management across different settings [5]. This case report highlights the challenges of managing a giant inguinal hernia with malrotation in a resource-limited setting.

## 2 Case presentation

A 55-year-old male patient who underwent hernia repair surgery for an indirect inguinal hernia 2 years back currently presented with a vast scrotal mass of 1 and a half years duration associated with intermittent **lower abdominal pain**. On examination, there was a surgical scar over the right inguinal region and significant irreducible, non-tender right inguinoscrotal swelling reaching up to the mid-point of the inner thigh. This work has been reported in line with the SCARE criteria [6].

**The patient was scheduled for elective surgery under spinal anesthesia**. An oblique inguinal incision was made, and subsequently, the sac was separated from the cord and opened; reduction of content from the scrotum was challenging, and terminal ilium, appendix, cecum, ascending colon, transverse colon, descending colon, and proximal part sigmoid colon was found to be herniated into the scrotum with no evidence of ischemia. By this time, laparotomy was done for adequate evaluation of the **mal-rotation and** proper replacement of the bowel. It was found that the ascending colon and descending colon did not have a retroperitoneal attachment. The third and fourth part of the duodenum descended vertically on the right side in the absence of ligament of Treitz. Ladd's procedure was done with appendectomy. The wound was closed primarily without undue tension; the patient had a smooth postoperative course and discharged on the 5th post-op day. Up on fellow up on his 14th post-op, he had no complaint, and the **wound was healing.**

**Fig. 1:**
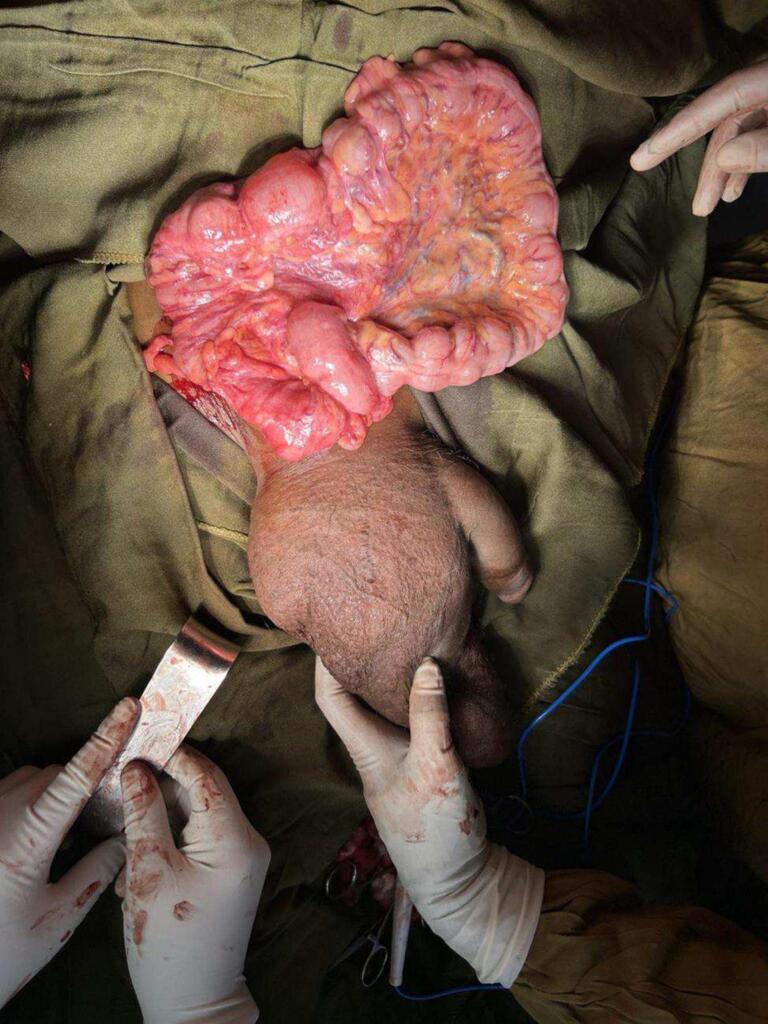
- shows intra-operative reduction of content from the scrotum through an oblique right inguinal incision (terminal ileum, cecum, ascending colon, transverse colon, descending colon, and proximal sigmoid colon) but i\still some distal sigmoid colon is inside the scrotum as shown on the image, thought the scrotum is partially decompressed it reaches up to mid-thigh.

See Fig. 1**:** - for how intra-operative reduction of content from the scrotum through an oblique right inguinal incision (terminal ileum, cecum, ascending colon, transverse colon, descending colon, and proximal sigmoid colon) but i\still some distal sigmoid colon is inside the scrotum as shown on the image, thought the scrotum is partially decompressed it reaches up to mid-thigh.

## 3 Discussion

Giant inguinoscrotal hernias (GIH) are classified into three types based on their extent, influencing management approaches. Type I hernias, which extend to the suprapatellar margin, generally require only hernioplasty with intra-abdominal and intrathoracic pressure monitoring without additional interventions to prevent abdominal compartment syndrome (ACS). Types II and III, characterized by loss of domain, may necessitate further measures to prevent ACS, such as resection of hernia contents and techniques to increase abdominal volume, including Preoperative Progressive Pneumoperitoneum (PPP), Botulinum toxin A injection and component separation. Studies report successful outcomes with these approaches, even in complex bilateral cases, highlighting the need for tailored management strategies based on hernia classification [7].

The development of giant inguinal hernias, despite advancements in medical procedures and the availability of effective treatments, remains a significant issue. Several factors contribute to this phenomenon. For instance, a case report from Kashmir, India, describes a 62-year-old male whose hernia developed primarily due to a lack of awareness regarding the surgical procedure. When the patient was informed about the possibility of local anesthesia, he had accepted the procedure. This highlights the critical role of patient education and awareness in the timely management of such conditions [8].

In another case from Nepal, a 65-year-old male presented with progressive swelling and multiple non-discharging ulcers over the scrotal skin. The patient delayed seeking medical attention due to financial constraints and a lack of confidence in the surgical system [4]. These factors, particularly financial limitations, play a significant role in the neglect of such conditions. A study investigating the reasons for delays in treatment found that financial barriers and neglect of the condition were the primary causes. Additionally, the patients often sought medical care only when their condition interfered with daily activities, such as the inability to engage in sexual intercourse, which led to psychological distress [9].

Giant inguinoscrotal hernias (GIH) pose significant challenges for patients and surgeons, particularly in resource-limited settings. These massive hernias can severely impact mobility, leading to difficulties in walking, sitting, or even lying down. Patients may also experience scrotal skin ulceration, urinary retention due to pressure on the penis, and potentially life-threatening tissue expansion of vascular pedicles [1] [10]. Our patient's case highlights the challenges of managing a GIH in a resource-limited setting, as he initially declined surgery due to the perceived complexity and potential complications.

Various surgical techniques have been described for GIH repair, with approaches focusing on either increasing abdominal capacity or reducing hernia contents. Preoperative progressive pneumoperitoneum therapy, involving inflating the abdominal cavity with air to create space for hernia contents, has shown promise in some cases but can lead to discomfort, shoulder pain, and potential complications [11] [12]. Another approach involves debulking the hernia contents by resecting bowel segments, which carries the risk of anastomotic insufficiency and prosthetic infection [13] [14]. The best surgical strategy must be tailored to the individual patient, their health, and available resources. A combined approach involving a transabdominal midline incision, omental resection, and mesh placement in a preperitoneal position successfully reduced the hernia without requiring bowel resection.

Minimally invasive surgery for giant inguinoscrotal hernias is challenging due to their size and potential for “loss of domain,” often requiring open approaches like the one described by Staubitz et al. Their case highlights the need for open surgery to address complex issues that may include component separation, tailored mesh placement, and orchiectomy after a failed laparoscopic attempt. While future possibilities may lie in improving abdominal space or robot-assisted techniques, open surgery, and combined open approaches, remains a necessary option, allowing for the best results as seen in the successful repair by the authors of the case study [15].

The presence of malrotation in our patient is unusual, as this congenital anomaly is typically diagnosed in infancy [16] [17]. This case highlights the importance of considering potential anatomical variations, even in adult patients with GIH. Complications of GIH repair are common and can include respiratory compromise, wound dehiscence, hematocele/seroma formation, and recurrent hernias. These complications can be more challenging to manage in resource-limited settings [18,19]. Careful postoperative follow-up is essential to monitor for complications and ensure long-term success. In our patient's case, the successful repair with the chosen surgical approach and meticulous postoperative care led to a favorable outcome. This case report can contribute to a better understanding of GIH management in resource-limited settings, promoting further research and encouraging the development of standardized protocols.

## 4 Conclusion

Giant inguinoscrotal hernias (GIH) represent a significant surgical challenge, particularly in resource-constrained environments. Their classification into types based on the extent of hernia provides a structured approach to management, with Type I hernias requiring simpler interventions and Types II and III often demanding advanced techniques to mitigate risks like abdominal compartment syndrome (ACS). Strategies such as Preoperative Progressive Pneumoperitoneum (PPP), botulinum toxin injections, and component separation demonstrate the importance of individualized, resource-aware surgical planning.

This case highlights the intricate decision-making required in managing GIH, especially in the presence of uncommon anatomical anomalies like malrotation. Successful outcomes can be achieved even in complex cases by combining careful preoperative planning, innovative surgical approaches, and meticulous postoperative care. Addressing these challenges in resource-limited settings emphasizes the need for ongoing research and the development of standardized management protocols to improve care for patients with GIH worldwide.

## Author contribution

All the authors in this work participated in the patient caring and treatment, conceptualization of the case report, drafted the manuscript, and reviewed the final version of the case report.

## Ethical approval

Ethical approval for this study was obtained.

## Guarantor

Dr. Hassan Sh. Abdirahman Elmi.

## Consent for publication

After thoroughly explaining the study's purpose and implications, the patient provided informed consent.

## Funding

No funding was received to conduct this study.

## Declaration of competing interest

There are no conflicts of interest related to the publication of this article.
